# Scalable Fabrication of Polyethylenimine (PEI) Doped Laser-Induced Graphene (LIG) Inks

**DOI:** 10.1021/acsomega.5c05259

**Published:** 2025-09-12

**Authors:** Fatmanur Kocaman Kabi̇l, Seda Kol, Nihan Aydemi̇r, Ahmet Yavuz Oral

**Affiliations:** † Institute of Nanotechnology, 52962 Gebze Technical University, Gebze, Kocaeli 41400, Türkiye; ‡ Department of Material Science and Engineering, Gebze Technical University, Gebze, Kocaeli 41400, Türkiye; § Nanotechnology Research Center (NUAM), Gebze Technical University, Gebze, Kocaeli 41400, Türkiye

## Abstract

Laser-induced graphene (LIG) is a scalable and cost-effective approach to fabricating carbon-based conductive materials for next-generation flexible electronics. Here, we report the synthesis of LIG by CO_2_ laser engraving of polyimide (PI) films, followed by doping with polyethylenimine (PEI). LIG powder was dispersed in a terpineol–ethanol–ethyl cellulose mixture, doped with 21.5 wt % PEI, and deposited as a thick film on a glass substrate using stencil-assisted application. Electrical conductivity measurements showed an increase from ∼20.8 S/m for LIG-only ink to 40 S/m after PEI doping, representing nearly a 2-fold enhancement attributed to increased electron carrier density and a Fermi level shift toward n-type behavior. Comprehensive characterization was performed to assess structural, morphological, and chemical modifications induced by doping. Scanning Electron Microscopy (SEM) confirmed the preservation of a porous, wrinkled graphene-like morphology. Energy-dispersive X-ray spectroscopy (EDS), X-ray photoelectron spectroscopy (XPS), and Fourier transform infrared spectroscopy (FTIR) showed the successful incorporation of nitrogen. Raman spectroscopy revealed increased defect density and a Fermi level shift characteristic of n-type doping, while X-ray Diffraction (XRD) analysis showed reduced crystallinity and the formation of an amorphous phase. This work demonstrates that PEI not only modifies LIG chemically but also provides structural versatility, highlighting its potential for integration into real-world applications such as flexible and printed electronics. The combination of laser-assisted synthesis and polymer doping offers a practical route to the development of carbon-based materials.

## Graphical abstract

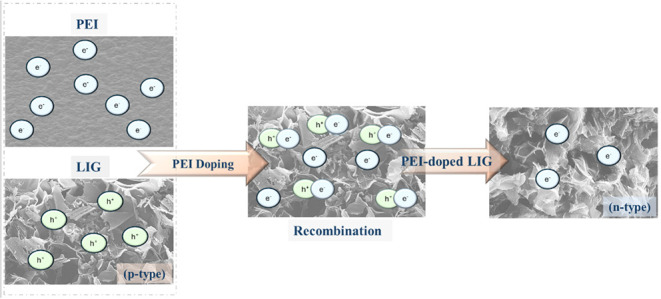

## 1 Introduction

Graphene is a well-known two-dimensional (2D) sp^2^ carbon material with a honeycomb structure.^1^ It exhibits outstanding mechanical, optical, and electronic properties, making it widely used in many applications such as transistors, energy storage, energy harvesting, and sensors.^2^ Graphene can be synthesized using various methods, including mechanical exfoliation, chemical exfoliation, chemical reduction, epitaxial growth, pyrolysis, chemical vapor deposition, and others.^3^ Laser-induced graphene (LIG) has recently emerged as an innovative technique, distinguishing itself from conventional methods through its high porosity and unique fabrication approach. LIG is a three-dimensional graphene material produced by exposing carbon-based materials to CO_2_ lasers in an atmospheric environment.^4^ The carbon-based polymer precursors are subjected to laser irradiation in order to undergo transformation into graphene, which is the mechanism that underlies this procedure. A temperature that is higher than 2500 °C is reached within the laser’s concentrated region as a result of the substrate absorbing the energy that is present in the laser.^4^ The intense heat generated by the laser causes the polymer to degrade, and its bonds (C–O, C–N, C=O, and C–H) break, leading to the formation of reactive intermediates and free radicals. Under high temperature, these species reorganize to form sp^2^-hybridized carbon bonds. This process facilitates the formation of graphene’s characteristic hexagonal lattice structure (Figure 1).^5^ Due to the existence of aromatic sp^2^ carbons, the chemical structure of polyimide (PI) is especially well-suited to produce LIG. This is because aromatic sp^2^ carbons are more likely to form the hexagonal graphene structure than other precursors, which only yield amorphous carbon.^6^ Additionally, the LIG powder can be obtained by scraping it off from the polyimide film for the preparation of various ink compositions used in printed electronics.^7^ The characteristics of graphene generated using LIG are influenced by variables like the selection of polymer precursor, laser power, scanning speed, wavelength, laser spot size, and the density of spots per inch (PPI).^8^

**Figure 1:**
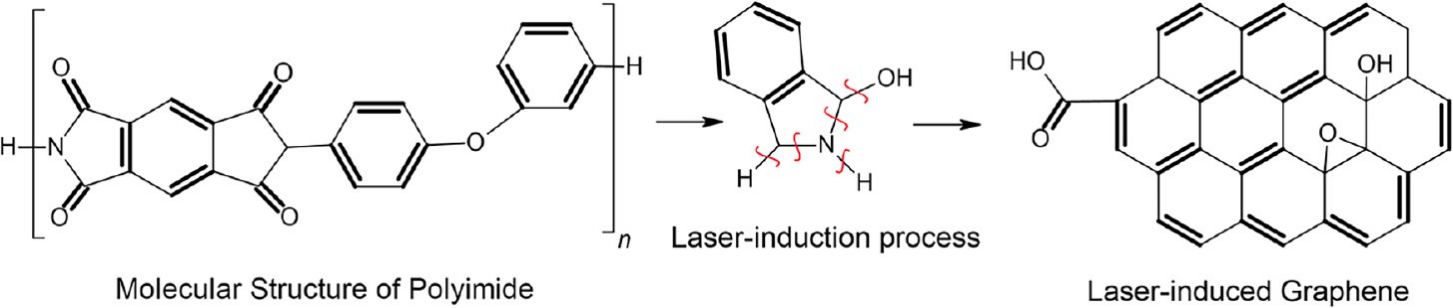
Laser-induced graphene formation from polyimide.

Unlike traditional graphene synthesis techniques, LIG enables the direct and mask-free patterning of conductive structures, while maintaining excellent material properties. LIG also exhibits high electrical conductivity, superior thermal stability, enhanced optical absorption, cost-effectiveness, and remarkable flexibility.^9^ Figure 1 shows the laser-induction process of PI and the corresponding photographs of the LIG films and scraped LIG powders. This technique provides a single-step approach for large-area patterning, eliminating the need for expensive cleanroom facilities, wet chemical processes, or subsequent treatments.^10^ Consequently, LIG has found applications in various fields, including energy harvesting devices,^11,12^ sensors,^13,14^ supercapacitors,^15,16^ and batteries.^17,18^

Doping is a highly effective and versatile technique for modifying graphene’s properties by increasing its carrier density and adjusting its work function.^19,20^ Electron injection into or extraction from graphene, a process known as n-type or p-type doping, is commonly used in Fermi level engineering.^21^ Substituting graphene with heteroatoms provides structural stability through covalent bonding, yet it disrupts its honeycomb lattice, introducing defects and disorder. The choice of a polymer as a dopant provides the benefit of uniform doping, which can generate a charge-neutral and low-disorder state, thereby enhancing carrier mobility.^22^

Organic amines are regarded as efficient n-type dopants for graphene, as their lone-pair electrons readily transfer to graphene through charge transfer.^23^ Polyethylenimine (PEI) is a polymer with amino groups that exists in two forms. While linear PEI consists solely of secondary amines, branched PEI contains primary, secondary, and tertiary amine groups.^24^ Notably, PEI can effectively donate electrons to graphene, which has a higher work function making it a suitable n-type dopant.^19^ A low work function is expected to create a significant energy barrier, preventing hole injection while promoting electron injection.^25^ An et al. utilized PEI as an n-type dopant to modify the electronic properties of graphene, aiming to enhance its performance in graphene-based supercapacitors.^22^ Wang et al. utilized PEI as an n-type dopant for graphene field-effect transistors (GFETs), resulting in a shift of the Dirac point from +54 to −22 V, confirming successful electron doping.^26^ Tu et al. utilized PEI as a surface charge transfer dopant to convert unintentionally p-type nitrogen-doped graphene derivatives into stable n-type materials by donating electrons. This p-type behavior was attributed to unintentional hole doping caused by adsorbed water and oxygen molecules, which PEI effectively counteracted. These findings demonstrate the potential of PEI doping in tuning the carrier properties of graphene-based thermoelectric devices.^27^

In this study, LIG was successfully n-type doped using PEI. Figure 2 illustrates the chemical structure of LIG subsequent to PEI doping. The schematic illustrates the covalent bonding mechanism between polyethylenimine (PEI) and the carboxyl groups (−COOH) on the surface of LIG. Amide bonds are known to form between the amine groups (−NH_2_) of PEI and the carboxyl groups of LIG, as commonly reported in the functionalization of graphene-based materials.^28,29^ In this mechanism, a covalent amide linkage (O=C–NH) is formed through a nucleophilic attack by the −NH_2_ group of PEI on the electrophilic carbon atom of the carboxyl group, indicating strong chemical immobilization of PEI onto the surface of LIG. A comprehensive characterization was conducted through Raman spectroscopy, Fourier-transform infrared spectroscopy (FTIR), X-ray photoelectron spectroscopy (XPS), X-ray diffraction (XRD), Scanning electron microscopy (SEM), and Energy-dispersive X-ray spectroscopy (EDS) to confirm the doping process and its effects on the material’s structure and composition. While previous research has reported graphene doping with PEI, no prior work has involved the preparation of viscous ink from LIG powder, followed by PEI doping and subsequent fabrication of conductive films. As a result, this work opens new horizons for the integration of doped LIG films into a broad spectrum of emerging applications, including thermoelectric devices, flexible and printed electronics, high-performance sensors, and next-generation energy systems.

**Figure 2:**
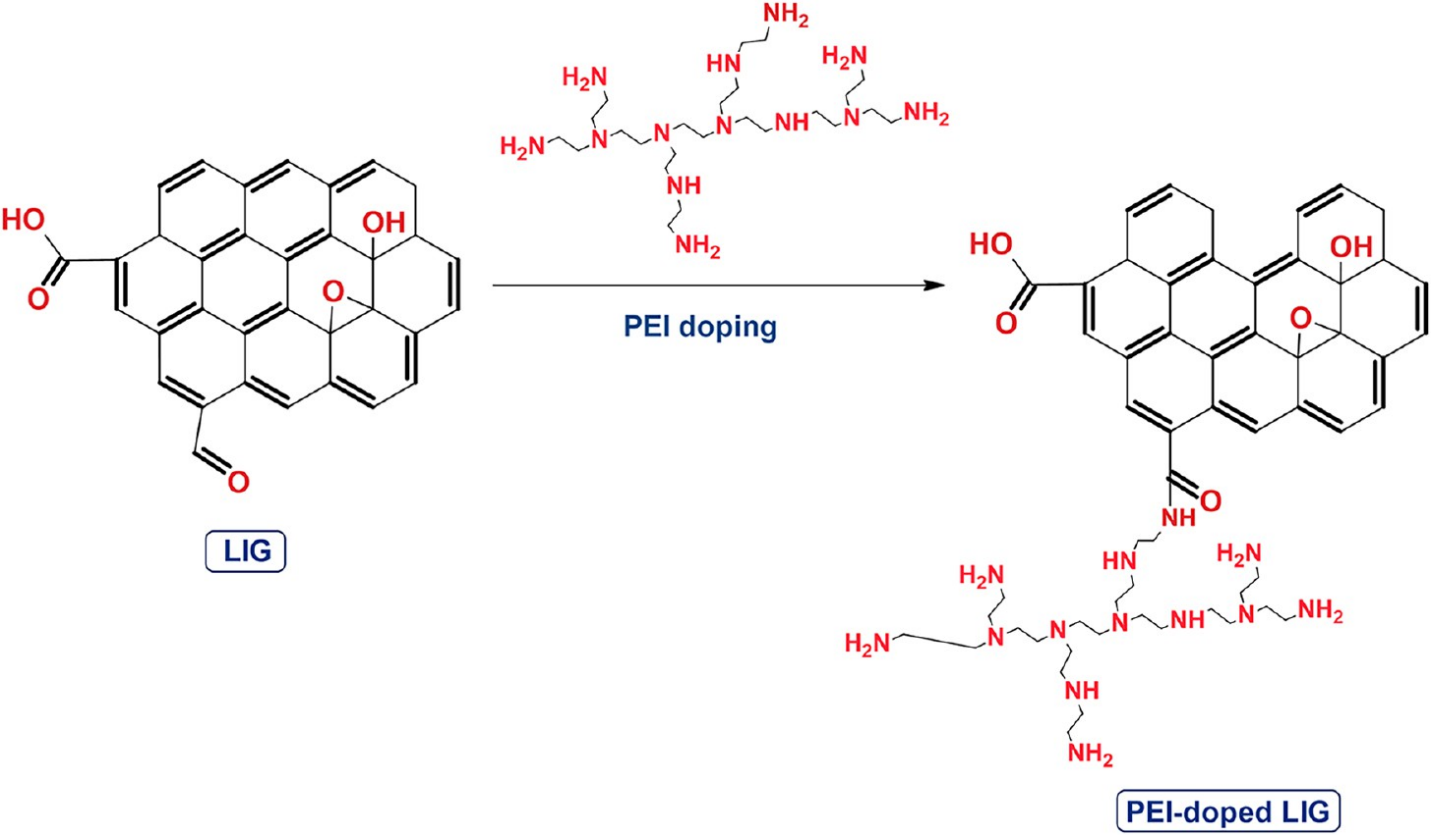
Chemical structure after PEI doping.

## 2 Experimental Studies

### 2.1 Chemicals and Materials

PEI (408727- branched), ethyl cellulose (EC) (247499- viscosity 100 cP, 5% in toluene/ethanol 80:20, 48% ethoxyl), terpineol (86480), and ethanol (100983, ≥99.9%) were purchased from Sigma-Aldrich (Merck). PI (Kapton Polyimide Film) with 100 μm thickness was used for the laser-induction process.

### 2.2 Fabrication of LIG Powder

LIG was fabricated by engraving a polyimide film using a CO_2_ laser with a wavelength of 10.6 μm. The laser system had a maximum power of 50 W, and LIG films were generated at 18.5% power with a scanning speed of 350 mm/s. After the LIG film was obtained, it was further processed by scraping to collect the LIG powder (Figure 3a). All laser experiments were conducted under ambient conditions. Figure 3b,c show photographs of both the fabricated film and the collected powder.

**Figure 3:**
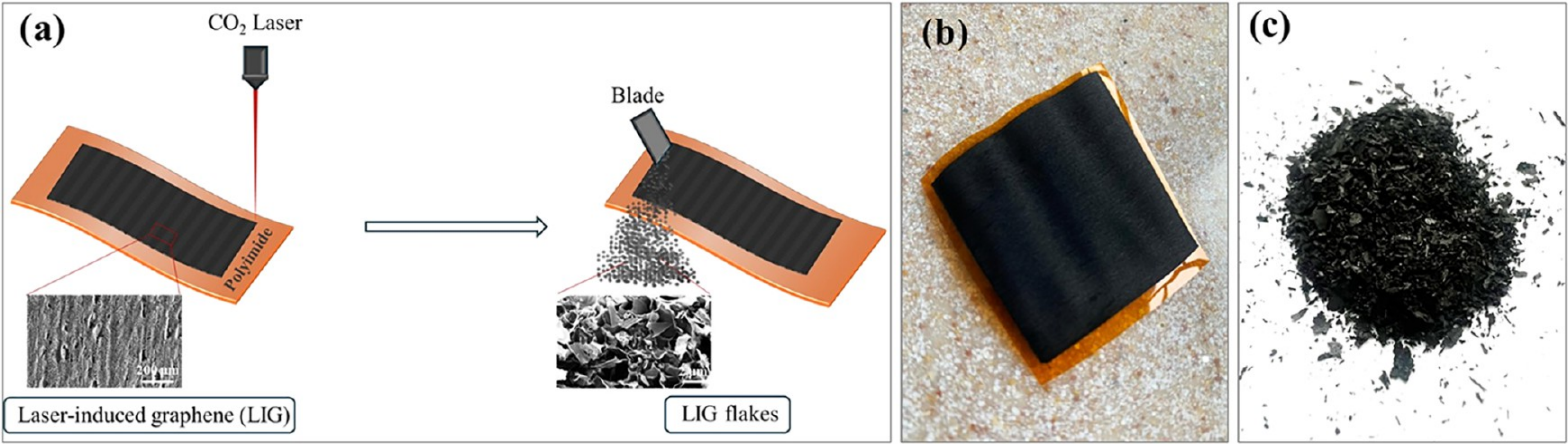
(a) Schematic illustration of LIG fabrication, (b) photo of the LIG film, and (c) LIG powders scraped off from polyimide.

### 2.3 Preparation of PEI-Doped LIG Film

PEI-doped graphene ink was prepared by dispersing LIG powder and ethyl cellulose (EC) in a terpineol/ethanol solvent mixture at a volume ratio of 1:5. EC is used as a binder to enhance the stability of the ink formulation while increasing its viscosity, thereby ensuring its suitability for printing applications. Terpineol, serving as a slow-evaporating solvent and carrier, facilitated the uniform dispersion of solid components and improved the rheological properties of the ink for a smooth and consistent application. Ethanol, employed as a cosolvent, temporarily reduced the viscosity of the ink, promoting better mixing and dispersion of the constituents before being completely evaporated during the drying process. Specifically, 80 mg of LIG and 120 mg of EC were added to 6 mL of the solvent mixture and subsequently stirred to ensure the initial dispersion. Next, PEI was added at a doping ratio of 21.5 wt %, calculated relative to the LIG content. To achieve a homogeneous dispersion, the mixture was subjected to ultrasonication, using a bath sonicator for 2 h. Following ultrasonication, the dispersion was transferred to a vacuum oven and dried at 70 °C until ethanol completely evaporated. A masking tape was applied to the glass substrate to create a stencil, and the ink was then deposited and spread by using a spatula to form a thick film. Figures 4 and 5 show the experimental procedures for the preparation of PEI-doped LIG conductive ink and a photo of the thick film formed on glass, respectively. The glass substrates were employed solely for characterization purposes as their fully amorphous nature prevents the appearance of any diffraction peaks that could overlap with reflections originating from the sample.

**Figure 4:**
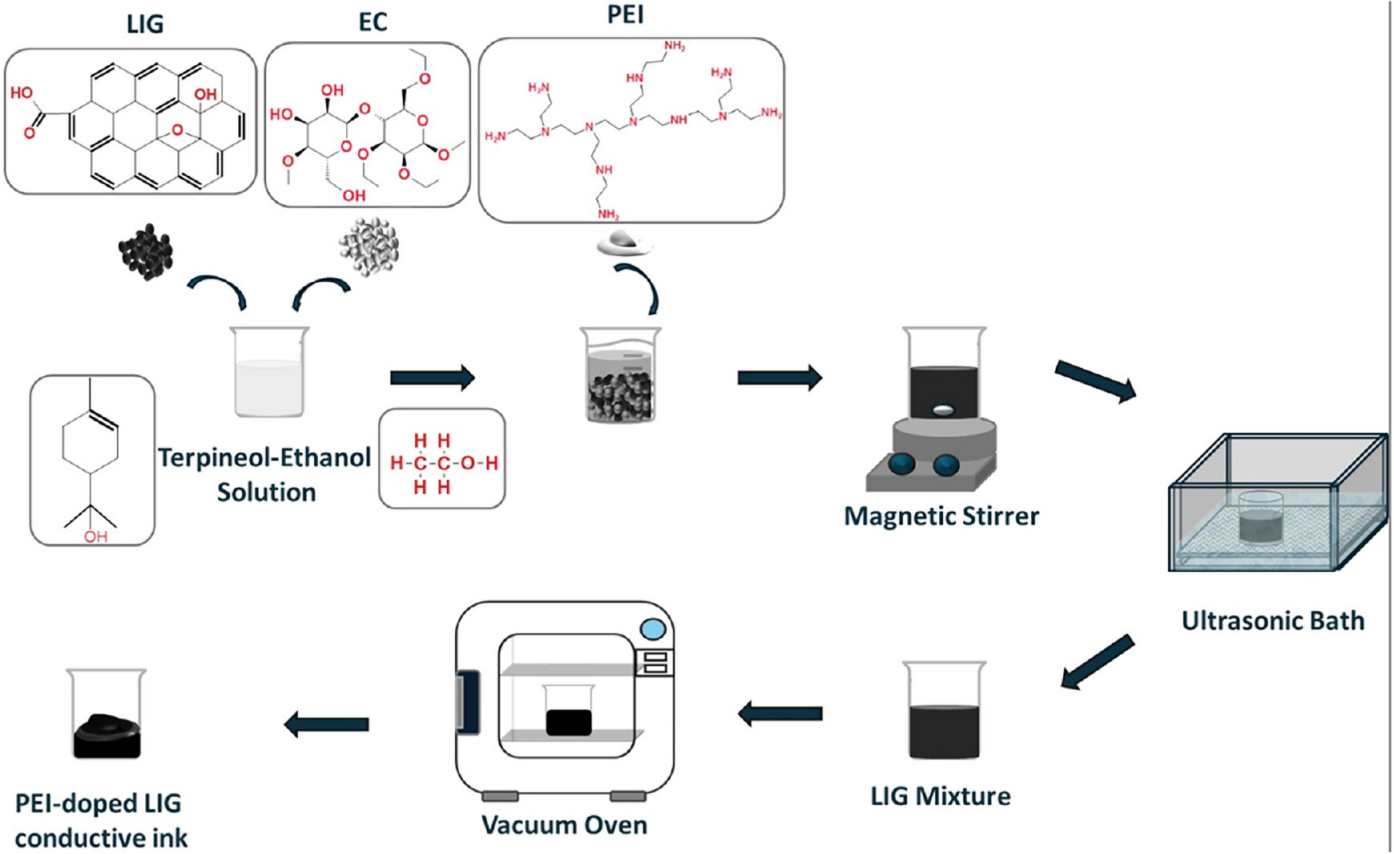
PEI-doped LIG conductive ink preparation steps.

**Figure 5:**
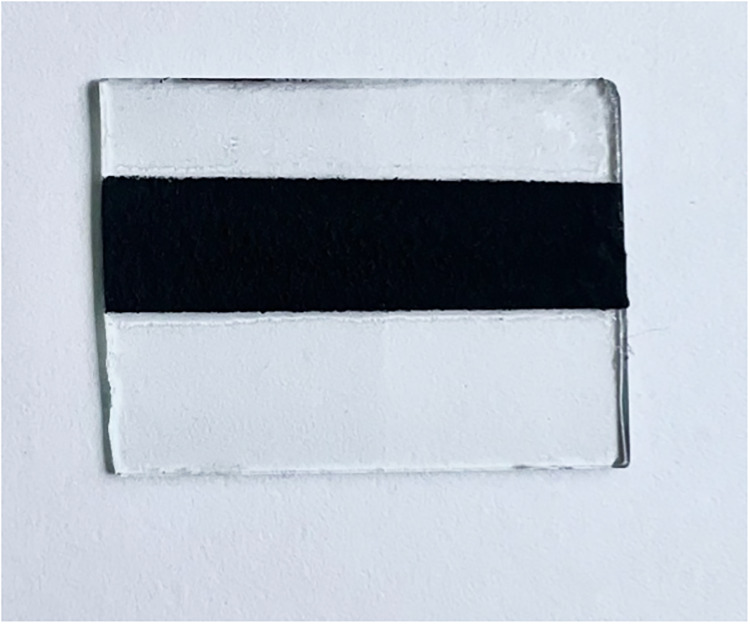
PEI-doped LIG thick film formed on a glass substrate.

## 3 Material Characterization

Scanning electron microscopy (SEM) images were obtained using a field emission SEM equipped with an energy dispersive X-ray spectrometer (SEM/EDS - Philips XL 30 SFEG-Ametek). The crystal structure of LIG and PEI-doped LIG was analyzed by X-ray diffraction (XRD, Rigaku Smartlab) with Cu–Kα radiation. X-ray Photoelectron Spectroscopy (XPS Phoibos 100, SPECS GmbH) was employed to assess the chemical composition of LIG, evaluate the efficiency of the PEI doping process, and identify the presence of functional groups. Fourier transform infrared (FTIR) spectra were collected by using a Bruker Tensor 27 FTIR spectrometer to investigate the chemical bonding and functional groups present in the samples. Raman spectra were acquired with a Renishaw Virsa Raman microscope by using 532 nm laser excitation at a power of 10 mW to study the vibrational modes and molecular structure of the materials. The electrical conductivities of the samples were determined by using a standard four-point probe method with a Keithley source meter.

## 4 Results

### 4.1 Surface Morphology Analysis

Surface morphology of the LIG film, LIG powder, and PEI-doped LIG film were characterized by scanning electron microscopy (SEM). In Figure 6a,b, SEM images of LIG films revealed that the surface exhibits a porous, sponge-like morphology, indicating a high surface area. This porous structure is attributed to the rapid release of gases generated during the irradiation of the polyimide substrate.^8^ Figure 6c,d presents SEM images of scraped LIG powders. The images reveal that the LIG powder exhibits an irregular and highly porous morphology, with many flakes displaying wrinkled or folded surfaces—typical characteristics of graphene-like structures. The flake sizes range from submicron to several microns (1–10 μm), indicating a polydisperse particle distribution.

**Figure 6:**
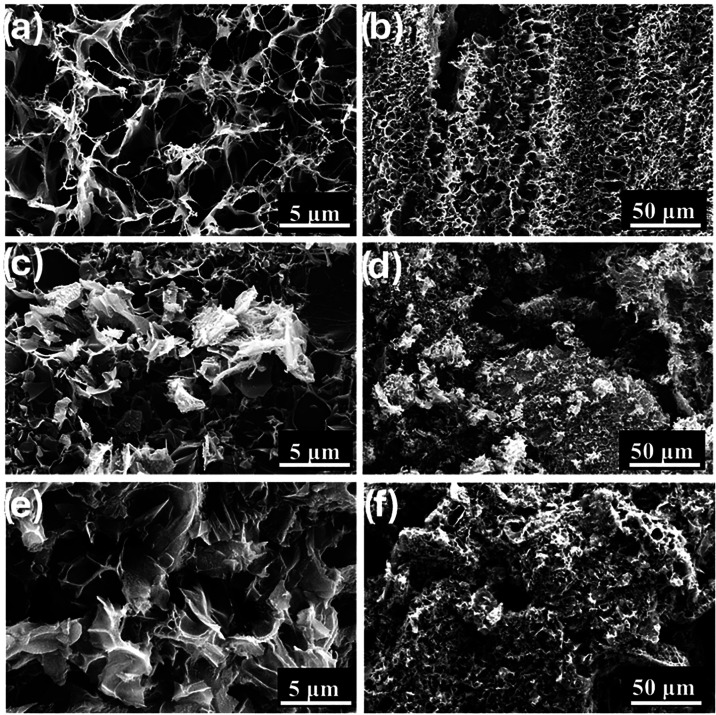
SEM images of (a, b) LIG film, (c, d) LIG powders, and (e, f) PEI-doped LIG with high and low magnifications.

Figure 6e shows a high-magnification SEM image of PEI-doped LIG, revealing a porous network composed of interconnected, wrinkled graphene-like sheets. This layered and crumpled morphology indicates that the PEI doping process preserves the intrinsic microstructure of LIG, which is beneficial for maintaining a high surface area and efficient charge transport pathways. The presence of thin sheet-like structures suggests good structural integrity and potential for applications requiring enhanced surface interactions.

Figure 6f displays a lower-magnification SEM image of the same PEI-doped LIG sample. The material exhibits a uniformly porous architecture with granular surface features, which contribute to surface roughening and enhance the material’s interfacial properties. The preserved porosity at this scale suggests suitability for applications such as sensing, energy storage, and flexible electronics, where both the surface area and conductivity are critical.

### 4.2 Chemical Composition Analysis

#### 4.2.1 EDS Analysis

An energy-dispersive spectroscopy (EDS) was employed to identify the chemical composition and elemental distribution of the pristine and PEI-doped LIG film.

Figure 7 displays the SEM image of the LIG surface alongside EDS spectra collected from four different regions of the sample, each indicated by colored boxes. The surface morphology appears highly porous, consistent with typical LIG structures derived from polyimide substrates.

**Figure 7:**
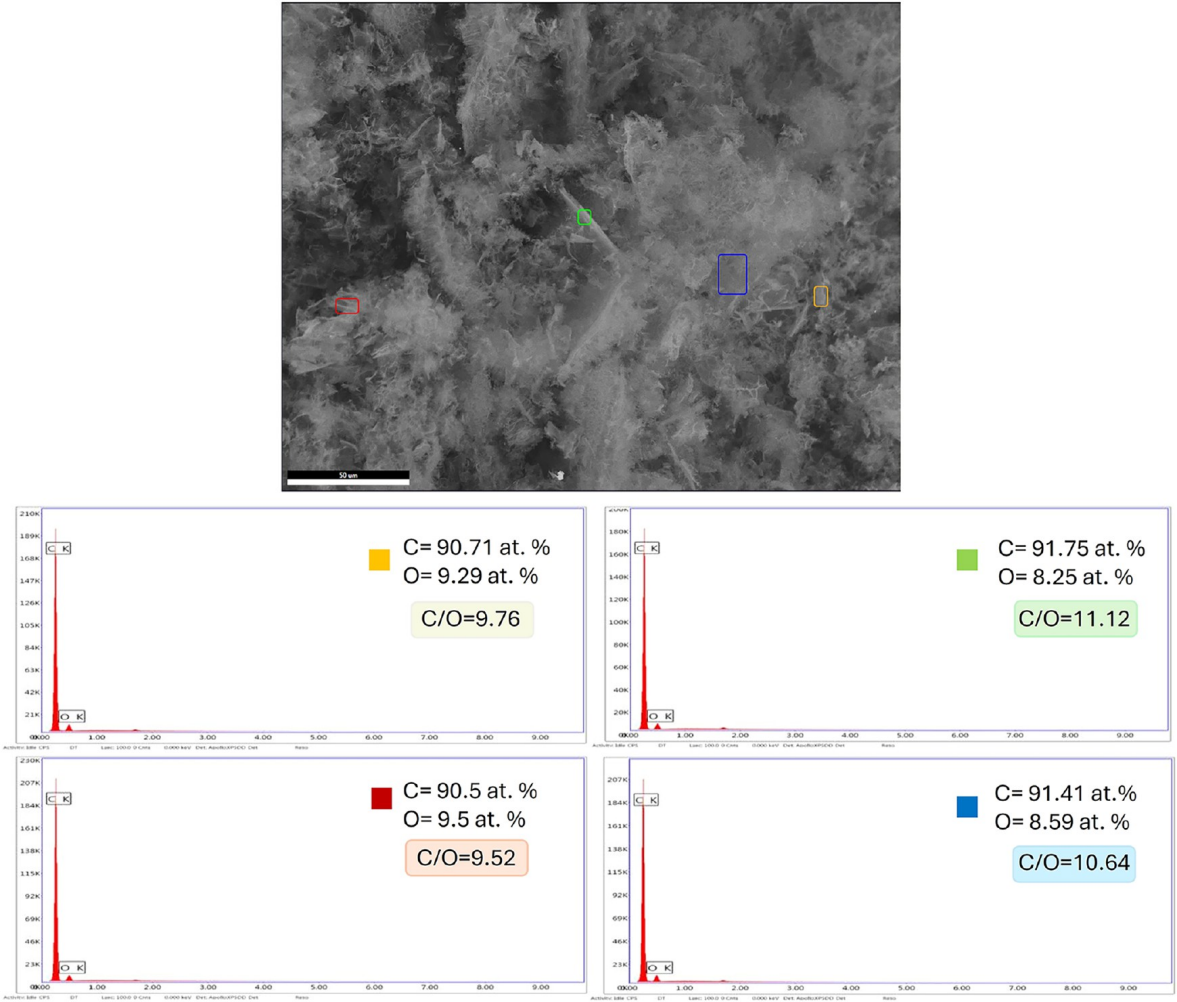
EDS analysis of pristine LIG.

The EDS spectra obtained from four different regions reveal that the LIG film is predominantly composed of carbon, with atomic percentages ranging from 90.5 to 91.75%. A significant amount of oxygen was also detected (8.25–9.5 atom %), which can be attributed to residual oxygen-containing functional groups or partial oxidation during the laser processing. The calculated atomic C/O ratios ranged from 9.52 to 11.12, indicating a high degree of carbonization of the precursor material. The absence of nitrogen in the EDS spectra indicates the transformation of the nitrogen-containing polyimide into a carbon- and oxygen-rich structure. The laser-induced graphitization process likely decomposed and removed most nitrogen species, leaving residual amounts below the EDS detection limit.

Figure 8 presents the SEM image, along with the corresponding EDS spectrum and elemental mapping results. The top-left SEM image reveals the porous surface morphology of the PEI-doped LIG film. The accompanying EDS elemental maps demonstrate the uniform distribution of carbon (C), nitrogen (N), and oxygen (O) across the film surface. The yellow (O), blue (C), and red (N) signals are homogeneously dispersed, indicating consistent elemental incorporation throughout the material. The absence of localized clusters or agglomerates suggests that the elements are well-distributed at the microscale, ensuring uniform electrical and chemical properties. It is noted that nitrogen originates solely from PEI, while oxygen is contributed by both LIG and EC. The presence and uniform distribution of nitrogen (N) strongly indicate a well-dispersed PEI structure within the LIG matrix, verifying the success of the doping process.

**Figure 8:**
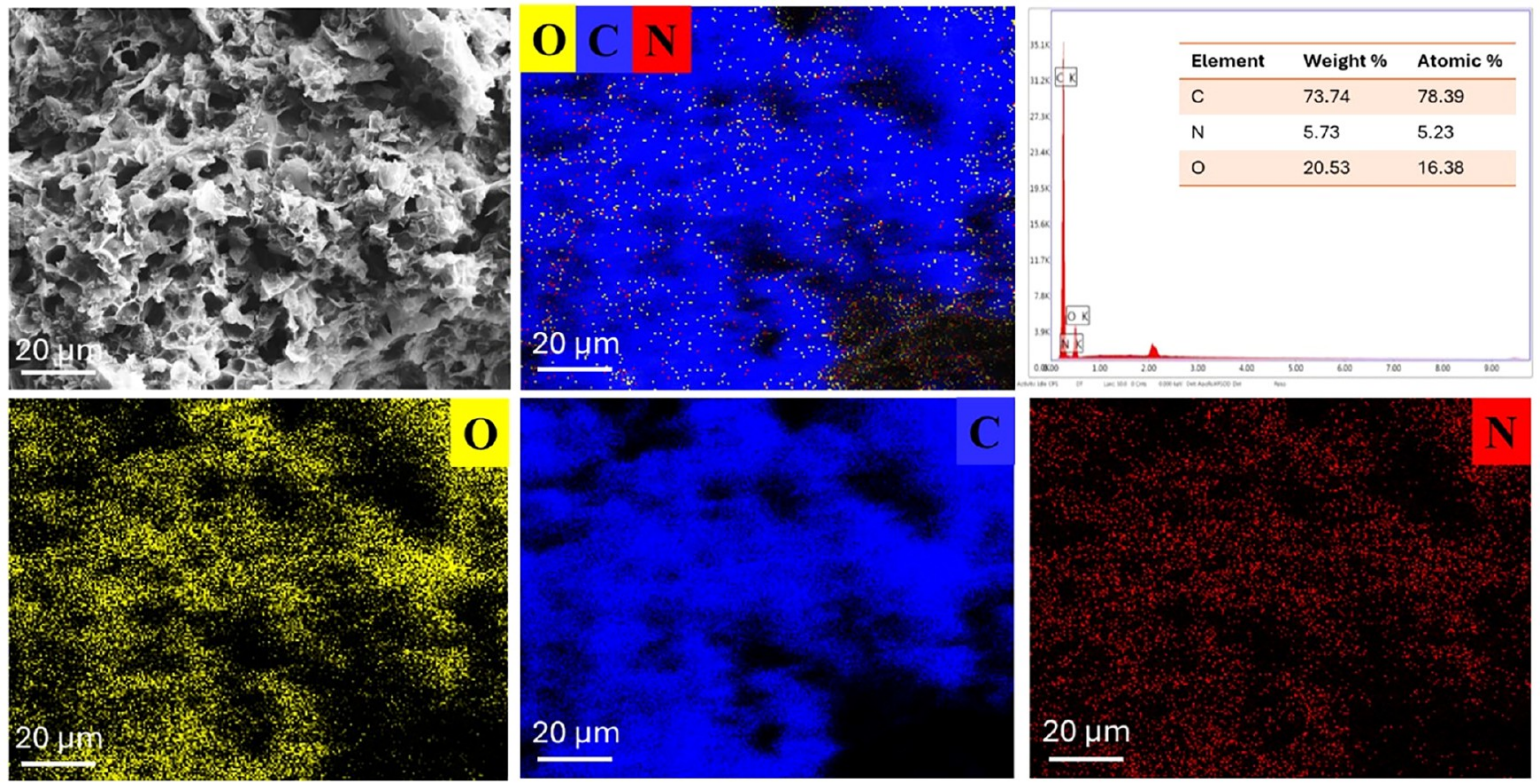
EDS elemental mapping of carbon (C), nitrogen (N), and oxygen (O) on the surface of the PEI-doped LIG film.

#### 4.2.2 XPS Analysis

The chemical compositions were analyzed using XPS with an Al Kα X-ray source (*hv* = 1486.6 eV) and a hemispherical electron analyzer (SPECS GmbH Phoibos 150). The chamber’s base pressure was 3 × 10^–10^ mbar throughout the analysis. The spectra were analyzed with CasaXPS, employing mixed Gaussian–Lorentzian peaks following the subtraction of a Shirley-type background to determine the chemical bonding energies.

The XPS survey spectra for the pristine LIG and PEI-doped LIG films were demonstrated in Figure 9. The total XPS spectra of pristine and PEI-doped LIG reveal that both materials exhibit notable characteristic peaks at 284.8 eV, corresponding to the C 1s peak, and an O 1s peak at around 533 eV. A distinct N 1s peak (approximately 400 eV) is observed in the full XPS spectrum of PEI-doped LIG, which is absent in the full XPS spectrum of LIG. Table 1 shows the atomic proportions of the three elements (C, N, and O) in the two samples. The higher oxygen content can be attributed to oxygen present in the EC structure, which was introduced during the doping process. Additionally, nitrogen (N), which was absent in pristine LIG, appeared in the doped sample with a concentration of 2.14%, confirming the successful incorporation of PEI.

**Figure 9:**
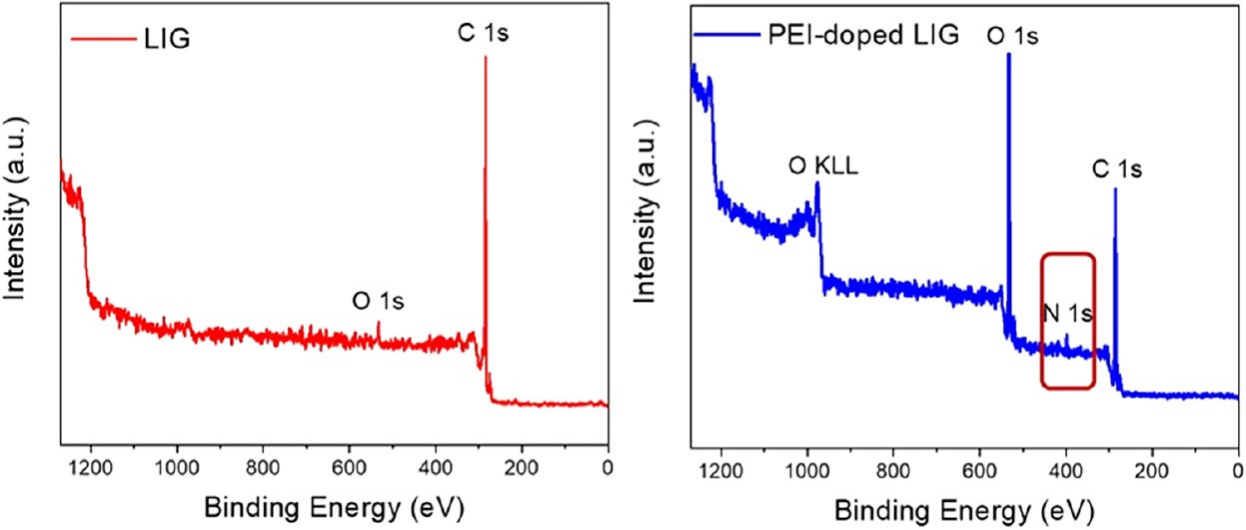
XPS survey analysis of LIG and PEI-doped LIG.

**Table 1:** XPS Surface Composition Analysis of LIG and PEI-Doped LIG Films

element	LIG (atom %)	PEI-doped LIG (atom %)
C	97.40	72.04
O	2.60	26.82
N	0	2.14

The C 1s, N 1s, and O 1s spectra of LIG and PEI-doped-LIG are subjected to curve fitting to investigate the element bonding configurations. The results are illustrated in Figure 10. All binding energies (BE) are referenced to adventitious C 1s (BE = 284.8 eV). The C 1s peak fitting results of LIG and PEI-doped LIG are illustrated in Figure 10a. The C–C peak is the prevalent peak in both LIG, suggesting that it was primarily composed of sp^2^ carbon. This outcome demonstrates that the substrate is effectively transformed to graphene through a laser-induction process. The analysis of the C 1s spectra reveals the presence of C–N and N–C=O bonds after PEI doping, indicating the successful incorporation of PEI into the structure. In particular, the transformation of O–C=O bonds into N–C=O further confirms the chemical interaction between PEI and LIG (Figure 2). Additionally, a decrease in the C–C/C=C peaks and an increase in C–OH signals were observed. The O 1s spectra indicate structural modifications, revealing the transformation between C–O and C=O bonds (Figure 10b), with the overall oxygen content originating from both LIG and EC. The observed increase in the C=O bond after PEI doping can be attributed to oxidative interactions occurring during processing. PEI and EC are organic polymers, and during processing—particularly during heating, drying, or solvent evaporation, they can come into contact with air and moisture. This exposure may trigger oxidation reactions. As a result, carbonyl (C=O) can reform on the surface of LIG), contributing to the increase in oxygen-related functional groups detected in the spectra.

**Figure 10:**
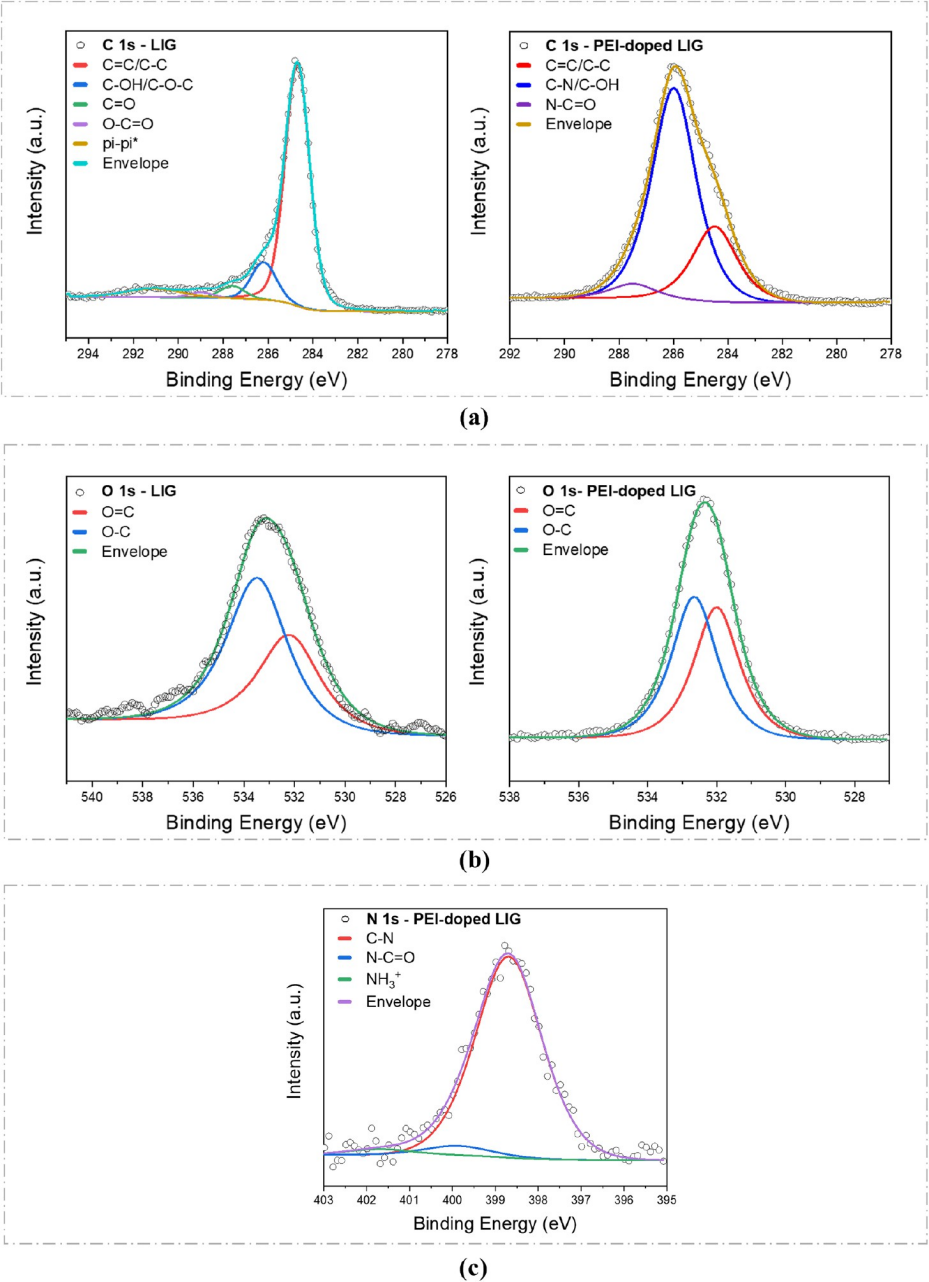
(a) C 1s, (b) O 1s, and (c) N 1s high-resolution XPS spectra of LIG and PEI-doped LIG.

As shown in the high-resolution N 1s spectrum in Figure 10c provides further insight into the nitrogen bonding environment in the PEI-doped LIG structure. The N 1s region can be deconvoluted into three distinct nitrogen species: C–N, N–C=O, and protonated amine (NH_3_^+^), all of which are associated with the chemical structure of PEI. These deconvoluted peaks confirm the successful incorporation of nitrogen-containing functional groups introduced by PEI during the doping process. The presence of these nitrogen states supports the proposed doping mechanism. Table 2 summarizes the atomic percentages and binding energies of the observed nitrogen species on the surface, providing quantitative validation of doping and surface functionalization. EDS analysis indicated approximately 5 atom % nitrogen, whereas XPS revealed around 2 atom %. This discrepancy arises from the fundamental differences in probing depth between the two techniques. EDS, with its greater probing depth (up to a few micrometers), captures the overall bulk nitrogen content, including subsurface regions. In contrast, XPS is a surface-sensitive technique with a probing depth of approximately 5–10 nm, and therefore only reflects the nitrogen concentration at the very surface. Surface oxidation may contribute to the lower nitrogen content observed by XPS. The formation of oxygen-rich groups (e.g., C=O, C–O) can hinder PEI binding or lead to the detachment of nitrogen-containing moieties, thereby reducing the detectable surface nitrogen signal.

**Table 2:** Binding Energies and Atomic Weights of All Species for LIG and PEI-Doped LIG

			atom %	
peak	species	binding energy (eV)	LIG	PEI-doped LIG
C 1s	C=C/C–C	284.69	74.39	18.01
	C–OH/C–O–C or C–N^a^	286.19	11.53	50.61
	C=O or N–C=O	287.59	3.81	3.41
	O–C=O	288.99	1.62	
	π-π	291.09	6.05	
	**total C%**		**97.40**	**72.03**
O 1s	C=O	532.19	1.02	12.44
	C–O	533.46	1.58	13.38
	**total O%**		**2.60**	**25.82**
N 1s	C–N	398.69		1.99
	N–C=O	399.89		0.10
	NH_3_	401.69		0.06
	**total N%**			**2.15**
	**total composition**		**100**	**100**

a Shows components specific to PEI-doped LIG.

### 4.3 Molecular and Structural Analysis

#### 4.3.1 FTIR Analysis

FTIR spectra of pristine LIG, pristine PEI, and PEI-doped LIG are presented in Figure 11, highlighting key functional groups. The pristine LIG spectrum (red) exhibits characteristic peaks corresponding to O–H stretching (∼3300 cm^–1^), C=C stretching (∼1600 cm^–1^), and CO_2_ adsorption bands (∼2300 cm^–1^). In contrast, the pristine PEI spectrum (black) is dominated by strong N–H stretching vibrations (∼3300 to 3400 cm^–1^), C–H stretching (∼2900 cm^–1^), and prominent C–N and N–H bending modes in the fingerprint region (∼1000 to 1500 cm^–1^).

**Figure 11:**
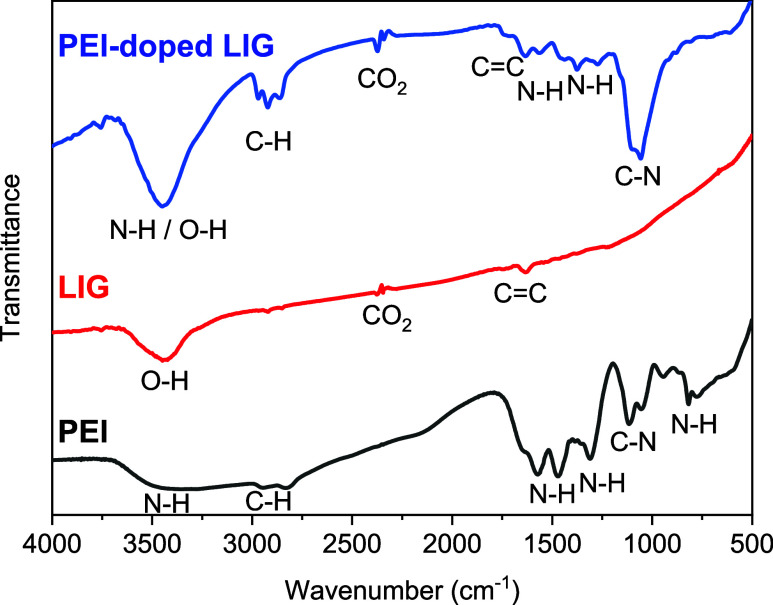
FTIR Spectra of LIG, PEI, and PEI-doped LIG.

Upon PEI doping, the PEI-doped LIG spectrum (blue) shows notable changes, confirming successful functionalization. The emergence of C–N (∼1200 cm^–1^) and intensified N–H (∼3300 cm^–1^) peaks indicates the incorporation of PEI into the LIG structure. Additionally, the shift and broadening of the N–H and C–H peaks suggest interactions between PEI and the LIG framework.

#### 4.3.2 Raman Spectroscopy Analysis

Raman spectroscopy was utilized to analyze the microstructures of the LIG and PEI-doped LIG film surfaces (Figure 12). Raman spectroscopy analysis of the pristine LIG film revealed a low *I*_D_/*I*_G_ ratio of 0.25, indicating a highly ordered graphene structure with minimal defects. Additionally, the *I*_2D_/*I*_G_ ratio of 0.63 suggests the presence of a multilayer graphene. Upon PEI doping, the *I*_D_/*I*_G_ ratio increased to 0.54, indicating a higher defect density and greater structural disorder, likely resulting from interactions between PEI and the graphene framework. Additionally, the decrease in the *I*_2D_/*I*_G_ ratio to 0.55 implies an increase in the number of graphene layers or the formation of more complex structural configurations. The Raman spectra of the PEI-doped LIG distinctly exhibit a *D*′ band at 1620 cm^–1^, which is ascribed to the intravalley double resonance scattering phenomenon.^30^ The Raman spectrum of PEI-doped LIG also exhibits a downshift of the G-band (2 cm^–1^) and the 2D-band (7 cm^–1^) compared to pristine LIG. Based on the empirical relationship between the Fermi energy and Raman peak position, these downshifts suggest that PEI dopants elevate the Fermi level of graphene.^31^ An increase in the Fermi level after doping indicates a shift toward n-type behavior, suggesting that the material has acquired additional electrons.

**Figure 12:**
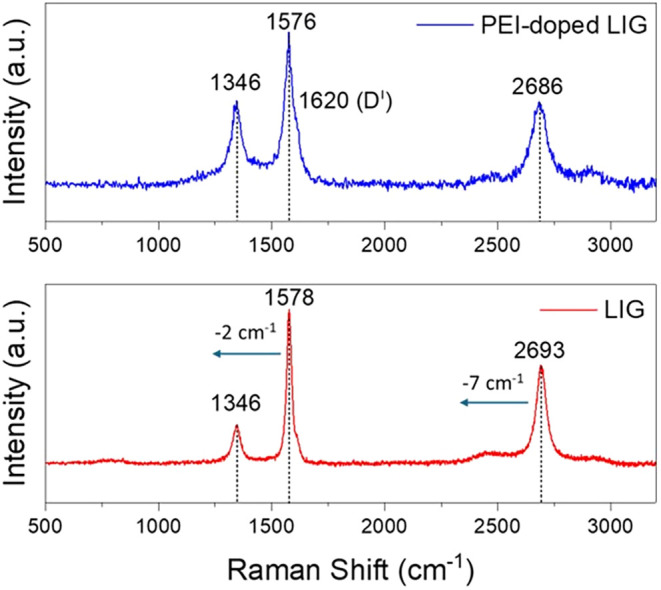
Raman spectra of LIG and PEI-doped LIG.

The electrical conductivity of the PEI-doped LIG film was also evaluated to gain insight into its functional performance. We prepared an ink using only LIG without PEI doping under identical formulation and processing conditions. The electrical conductivity of this LIG-only ink film was measured to be approximately 20.8 S/m, while the PEI-doped LIG ink film exhibited a conductivity of 40 S/m, representing nearly a 2-fold increase. This enhancement can be attributed to the increase in electron carrier density induced by PEI doping, which shifts the Fermi level away from the Dirac point, thereby reducing resistance and resulting in higher electrical conductivity.^19,32^ This behavior is consistent with the intrinsic nature of LIG, which typically exhibits p-type characteristics due to oxygen-containing functional groups and structural defects. Upon the introduction of PEI, the p-type holes are compensated by the amine groups in PEI, which act as electron donors. This results in a shift in carrier type to n-type by introducing excess electrons into the system. This transition from p- to n-type conductivity is further supported by the Raman spectroscopy results, which show an upward shift in the Fermi energy level, indicating an increased electron concentration due to effective n-type doping. Despite the reduction, the measured conductivity remains comparable to or better than many graphene-based composites reported in the literature.^33,34^

### 4.4 Crystal Structure Analysis

Figure 13 illustrates the X-ray diffraction patterns of LIG and PEI-doped LIG. Both exhibit distinct peaks at 2θ = 26° and 2θ = 43°, corresponding to the (002) and (100) crystal planes of graphene, respectively. It demonstrates that both exhibit the distinctive properties of graphene. The XRD pattern of PEI-doped LIG reveals important structural changes resulting from the doping process. Notably, the decrease in the sharpness of the (002) peak indicates a reduction in the crystallinity of the graphene domains, suggesting that PEI doping has introduced structural defects or disordered stacking within the graphitic layers.^35^ In addition, the incorporation of PEI leads to the appearance of a broad amorphous phase, alongside the main graphene peak. This outcome is expected, as the polymer chains interfere with the graphitic ordering and contribute to the formation of noncrystalline, amorphous regions in the overall composite structure.

**Figure 13:**
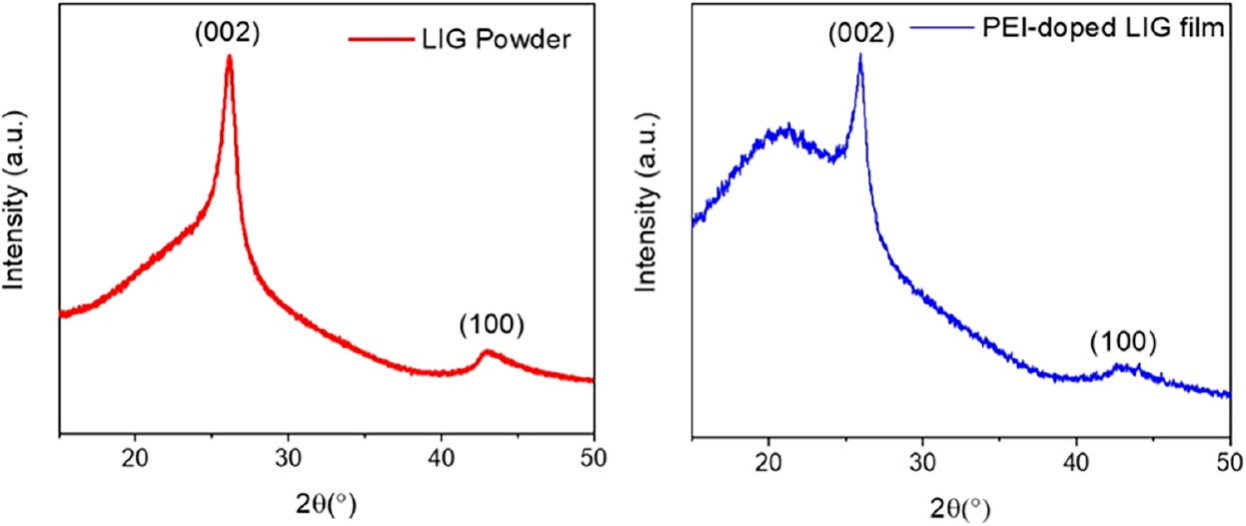
X-ray diffraction patterns of LIG and PEI-doped LIG.

## 5 Conclusions

In this study, LIG was successfully fabricated from polyimide films by CO_2_ laser engraving and then doped with PEI to produce a conductive composite ink. The PEI-doped LIG film retained the porous and wrinkled microstructure of the original LIG, as confirmed by SEM, which is beneficial for increasing the surface area and facilitating efficient charge transport. Elemental analysis by EDS and XPS confirmed the successful incorporation of the nitrogen-containing groups introduced by PEI. Furthermore, high-resolution XPS spectra showed the formation of C–N, N–C=O, and NH_3_^+^ bonds, indicating strong chemical interactions between PEI and the graphene matrix. The appearance of a C–N peak (∼1200 cm^–1^) and the intensified N–H stretching band (∼3300 cm^–1^) in the FTIR spectra, along with the shift and broadening of the N–H and C–H peaks, support the successful integration of PEI into the LIG structure and indicate significant molecular interactions within the framework. Raman spectroscopy further supported this transition by revealing an increased level of structural disorder and an upward shift in the Fermi level, both of which are consistent with n-type doping behavior. Electrical conductivity measurements indicated values of ∼20.8 S/m for the LIG-only ink and 40 S/m after PEI doping, confirming effective carrier type modulation toward n-type behavior. In addition, XRD analysis showed a broadening and decrease in the intensity of the (002) peak and the appearance of an amorphous phase, confirming a decrease in the crystallinity due to polymer doping. These results show that PEI doping not only changes the chemical and electronic properties of LIG but also confers tunable structural features, making it a promising candidate for future applications in flexible electronics, sensors, and energy storage and generation systems. Future studies could focus on evaluating the performance of the material in real applications and investigating the impact of alternative dopants on the overall functionality of LIG-based materials. Additionally, future studies will focus on printing the PEI-doped LIG ink onto various flexible substrates to evaluate its mechanical flexibility, adhesion properties, and durability under bending or stretching conditions. Such investigations will be crucial for assessing the material’s practical applicability in real-world wearable devices, flexible sensors, and energy harvesting systems, where substrate compatibility and mechanical stability are essential for reliable device performance.
